# The Uniform System of Accounts

**Published:** 1920-02-21

**Authors:** J. Danvers Power

**Affiliations:** 7 Ashley Gardens, S.W.


### THE UNIFORM SYSTEM OF ACCOUNTS.

The two first following letters appeared in *The Times* of February 14 and 17 respectively.

### HOSPITAL ACCOUNTS.

To the Editor of *The Times*.

Sir,—It is quite true that the original uniform system of hospital accounts was designed by Sir Henry Burdett; but his system was of an elementary character and not completely adapted for purposes of statistical comparison. What happened was this: A statistical report on hospital expenditure for 1903, based on accounts rendered under Sir Henry Burdett's system, was prepared by King Edward's Hospital Fund; and his present Majesty, then President of the Fund, called public attention to the wide variations in expenditure which it disclosed. The hospital secretaries, however, pointed out that the system of accounts required revision before perfectly satisfactory comparisons could be made, and nominated a committee which, under the chairmanship and competent professional advice of the distinguished accountant Mr. J. G. Griffiths, produced a revised system and form of balance sheet. These were adopted by King Edward's Hospital Fund, and, with a few subsequent amendments, remain in force. The Statistical Report has been issued annually since 1903, and has been the means of saving sums varying from £40,000 to £50,000 a year for the London hospitals alone. Indeed, I think I might say that, as Prince of Wales, His Majesty's policy in this matter was equivalent to an endowment for the London hospitals of a sum equal to a million.

I can imagine no more suitable and useful service for the Red Cross in time of peace than similar work for the provincial hospitals.

Your obedient servant,
J. Danvers Power.

7 Ashley Gardens, S.W.

